# Lipid‐Lowering Trials Are Not Representative of Patients Managed in Clinical Practice: A Systematic Review and Meta‐Analysis of Exclusion Criteria

**DOI:** 10.1161/JAHA.122.026551

**Published:** 2022-12-24

**Authors:** Martina Aeschbacher‐Germann, Nathalie Kaiser, Alexandre Speierer, Manuel R. Blum, Douglas C. Bauer, Cinzia Del Giovane, Drahomir Aujesky, Baris Gencer, Nicolas Rodondi, Elisavet Moutzouri

**Affiliations:** ^1^ Department of General Internal Medicine, Inselspital Bern University Hospital, University of Bern Switzerland; ^2^ Institute of Primary Health Care (BIHAM) University of Bern Switzerland; ^3^ Departments of Medicine and Epidemiology and Biostatistics University of California San Francisco CA; ^4^ Division of Cardiology Geneva University Hospitals Geneva Switzerland

**Keywords:** exclusion criteria, external validity, generalizability, lipid trials, multimorbidity, statins, Lipids and Cholesterol, Quality and Outcomes, Cardiovascular Disease, Women, Vascular Disease

## Abstract

**Background:**

Randomized clinical trials (RCTs) might not be representative of the real‐world population because of unreasonable exclusion criteria. We sought to determine which groups of patients are excluded from RCTs that included lipid‐lowering therapy.

**Methods and Results:**

We retrieved all trials from the Cholesterol Treatment Trialists Collaboration and systematically searched for large (≥1000 participants) lipid‐lowering therapy RCTs, defined as statins, ezetimibe, and PCSK9 inhibitors. We predefined groups: older adults (>70 or >75 years), women, non‐Whites, chronic kidney failure, heart failure, immunosuppression, cancer, dementia, treated thyroid disease, chronic obstructive pulmonary disease, mental illness, atrial fibrillation, multimorbidity (≥2 chronic diseases), and polypharmacy. We counted the number of RCTs excluding patients of the predefined groups and meta‐analyzed the prevalence of included patients to obtain pooled estimates with a random‐effects model. We included 42 RCTs (298 605 patients). Eighty‐one percent of trials excluded patients with severe and 76% those with moderate kidney failure. Seventy‐one percent of trials excluded groups of women, 64% excluded patients with moderate to severe heart failure, 64% those with immunosuppressant conditions, 48% those with cancer, 29% those with dementia, and 29% of trials excluded older adults. The pooled prevalence for patients >70 years of age was 25% (95% CI, 0%–49%), 11% (3%–18%) for >75 years of age, and 51% (38%–63%) for multimorbidity.

**Conclusions:**

The majority of lipid‐lowering therapy trials excluded patients with common diseases, such as moderate‐to‐severe kidney disease or heart failure or with immunosuppression. Underrepresenting certain populations, including women and older adults, might lead to limited transportability of study results and uncertainty on possible side‐effects and efficacy in these groups. Future trials should promote diversity in the recruitment strategies and improve equity in cardiovascular research.

**Registration:**

URL: ClinicalTrials.gov; Unique Identifier: CRD42021253909.

**Nonstandard Abbreviations and Acronyms**
CTTC: Cholesterol Treatment Trialists Collaboration
LLT: lipid‐lowering therapy

**Clinical Perspective**

**What Is New?**

- Patients with conditions such as severe renal dysfunction (creatinine clearance <30 mL/min), severe congestive heart failure, immunosuppressant conditions, dementia, and mental illness were often excluded in large lipid‐lowering therapy trials, while more than two‐thirds of all studies excluded groups of women.
- One‐third of the trials excluded patients >75 years of age, with a pooled prevalence of elderly patients (>75 years of age) of only 11%, while no trial, even recent trials, listed multimorbidity or polypharmacy as exclusion or inclusion criterion, with a pooled prevalence of multimorbid patients (defined as ≥2 chronic diseases) of 51% (95% CI, 38%–63%), suggesting that we need more trials that specifically target these populations.

**What Are the Clinical Implications?**

- Most lipid‐lowering trials excluded large groups of patients managed in clinical practice, which could possibly lead to uncertainty in treating these groups, while exclusion of groups of women may contribute to limited knowledge of the efficacy and safety of lipid‐lowering therapy in women compared with men.

Mortality from cardiovascular disease (CVD) is high, causing 8.9 million deaths worldwide by 2019,^1^ ≈45% of all deaths in Europe and 16% globally.^2^ Primary and secondary CVD prevention measures, such as lifestyle changes or treatment with lipid‐lowering medication, can reduce CVD mortality. Guidelines widely recommend statins as secondary prevention.^3^ However, their benefits and harms are less clear for primary prevention, because the net benefit of statins on absolute risk reduction depends on the individual's baseline risk of CVD,^3^, ^4^, ^5^ and their use is more controversial.^6^

Evidence of the effects of statins on CVD risk might have been drawn from trials with selected patient groups that do not represent real‐world demographics, as previously observed in RCTs in other research fields.^7^, ^8^, ^9^, ^10^ Compared with community populations, RCTs less often represent patients with multimorbidity or older age, and these patient groups are often excluded from RCTs.^11^, ^12^ Likewise, people with specific concomitant chronic diseases are commonly excluded.^13^ In addition, 1 study found that trials conducted between 2005 and 2015 investigating heart failure (HF), coronary artery disease, or acute coronary syndrome underrepresented women and found that sex‐related differences may have a relevant impact on drug efficacy and safety.^14^ Exclusion of specific groups might explain possible differences between observed rates of adverse effects in clinical practice compared with RCTs but might also explain possible different outcomes in certain populations.^15^

Limited data suggest that some groups of patients might have been excluded in early statin trials.^9^, ^16^ Moreover, there is little evidence on the efficacy of statin treatment in the multimorbid, elderly population or those with polypharmacy.^17^, ^18^, ^19^, ^20^, ^21^ It is therefore crucial to investigate which patients were included in recent large lipid‐lowering RCTs. Therefore, we performed a systematic review and a meta‐analysis to assess the prevalence of different patient groups in these trials.^22^

## METHODS

### Data Availability

Extracted data are available upon request to the corresponding author.

This systematic review followed a prespecified, published protocol and adhered to PRISMA (preferred reporting items for systematic review and meta‐analysis protocols) guidelines.^23^ Prospero registration: CRD42021253909.

### Search Strategy and Selection Criteria

The 2019 meta‐analysis of the CTTC (Cholesterol Treatment Trialists Collaboration) served as our baseline study. We retrieved all eligible studies mentioned on the homepage until May 2021.^24^ To update the search, we searched Medline, Embase, and Cochrane Central for large (≥1000 participants) lipid‐lowering RCTs published in English between January 1, 2015 and May 25, 2021 (Data S1). All trials with cardiovascular outcomes and a minimum 2‐year follow‐up were considered. We limited the search to the last 6 years (January 2015–May 25, 2021), because the CTTC updated their last meta‐analysis^21^ on large statin trials until this date, with the latest included trial published in 2016. For nonstatin trials, the first published trial on cardiovascular outcomes was published in 2015.^25^ For ezetimibe, the landmark trial on cardiovascular outcomes is IMPROVE‐IT (The Improved Reduction of Outcomes: Vytroin Efficacy International Trial),^26^ published in 2015.

We defined lipid‐lowering trials as trials with drugs that are known to reduce cardiovascular risk (statins, ezetimibe, and proprotein convertase subtilisin kexin type 9 inhibitors). We restricted our review to randomized controlled trials with participants ≥18 years of age who received lipid‐lowering drugs for primary or secondary prevention of CVD, with CVD and/or mortality as primary outcomes. We included all RCTs that compared a statin, ezetimibe, a proprotein convertase subtilisin kexin type 9 inhibitor or a combination of these drugs with one of the following: placebo, usual care, no treatment, another lipid‐lowering medication, or different dose. We excluded trials that did not report a cardiovascular primary outcome, trials that included duplicate data (eg, substudies of an already included original article), secondary analysis of subgroups of trials, post‐trial follow‐up studies, and ongoing trials. The supplemental index contains a full list of search terms and our search strategy (Data S1).

Two review authors (M.A. and A.S.) independently screened each study's title and abstract for eligibility. Then M.A. and A.S. independently screened the full text of potentially eligible studies, applied our exclusion list (Figure S1), and resolved disagreements through discussion. No third opinion was needed. When 1 study generated multiple publications, we included the main paper from the trial, which contained the most relevant data. If the main study referenced a separate publication describing baseline characteristics or study design, we included both publications, counted as 1 study. Because the present study was a meta‐analysis and not an intervention study, no ethical approval or informed consent was required, as per the Declaration of Helsinki.

### Data Collection and Management

One author (M.A.) extracted the data to an Excel spreadsheet, and a second reviewer (A.S.) independently checked the data, resolving disagreement by discussion. We extracted data on publication details (study name, author, and year of publication), characteristics of the study population (number of participants, primary or secondary prevention, baseline characteristics, demographics, inclusion and exclusion criteria, and prevalence of medical conditions and medication), characteristics of the intervention and the comparison groups (type and dosage of lipid‐lowering medication or placebo, and comparison groups), and the study's primary outcome. We did not contact study investigators to ask for unreported data.

M.A. and A.S. used the Cochrane Risk of Bias 2 tool to assess the risk of bias for each study included in the analysis. Cochrane Risk of Bias 2 is a tool for randomized controlled trials structured into 5 domains of bias, focusing on different aspects of trial design, conduct, and reporting (bias because of allocation concealment and failures of randomization, bias because of deviations from intended interventions, bias because of missing outcome data, bias in measurement of the outcome, and bias in selection of the reported result). Based on the answers to the signaling questions, an algorithm judges the domains as “low,” “some concerns,” or “high risk” of bias.^27^

We predefined the following patient groups: older adults (≥70 or ≥75 years of age), women, non‐Whites, chronic kidney disease (CKD) defined as estimated glomerular filtration rate <45 mL/min (moderate) or estimated glomerular filtration rate <30 mL/min (severe), HF according to New York Heart Association classification, immunosuppressant conditions, cancer, dementia, treated thyroid disease, chronic obstructive pulmonary disease, mental illness, atrial fibrillation, multimorbidity, and patients with polypharmacy. Because of the reporting of the age prevalence, we had to change our definition from ≥70/75 years of age to >70/75 years of age. We defined multimorbidity as ≥2 chronic conditions and polypharmacy as use of ≥5 drugs. Because most studies did not specifically report multimorbidity, we defined them by proxy. We gathered the prevalence data from the baseline table. If it was a secondary prevention trial, we assumed that 100% had the mentioned condition (we did not count hypercholesterolemia as a medical condition). We then checked the prevalence of the other mentioned conditions in the baseline table and assumed the minimum percentage of multimorbid patients by using the medical condition with the highest prevalence (ie, the minimum of multimorbid patients). If it was a primary prevention trial, we checked the baseline table for the prevalence of the mentioned conditions. If there were 2 conditions with a prevalence >50%, we assumed that the percentage >50% must be the minimum amount of multimorbid patients. We set our limit at 50% (not 60% as defined in the protocol) to avoid underestimating the amount of multimorbid patients. We did not count the amount of mentioned chronic comorbidities and divide it by the amount of patients as described in the protocol, because it would overestimate the amount of multimorbid patients because diseases are not evenly distributed.

### Data Synthesis and Analysis

We summarized baseline characteristics and inclusion and exclusion criteria in text and tables.

For each patient group, we calculated the percentage of studies that excluded the specific predefined group based on these criteria and baseline characteristics.

Because trials used different inclusion and exclusion criteria and defined groups differently, we categorized exclusion into 3 different subgroups. Trials that used clearly defined exclusion criteria for a specific patient group were classed as “*clearly excluded*.” Trials that used clearly defined exclusion criteria to exclude a segment of a patient group were classed as *“partially excluded”* (eg, only participants with HF New York Heart Association III‐IV were excluded). Trials that did not describe a specific patient group but circumscribed a medical condition were classed as *“probably excluded*” (eg, if a trial mentioned “other suspected serious physical illness” we assumed that they excluded patients with cancer or terminal renal failure).

When enough data were available, we meta‐analyzed the prevalence of specific patient groups that were included in the trials. For the quantitative synthesis, we used a random effects model to meta‐analyze the prevalence of these predefined groups across included studies; we transformed the data with Freeman‐Tukey and, for each group, estimated the pooled prevalence with a 95% CI.^28^, ^29^ Apart from the meta‐analysis based on the Freeman‐Tukey method, which provides a pooled prevalence in studies, called “the average rates” (ie, estimates the mean of the mean prevalence across studies), which has high heterogeneity because of difference in size between studies, we have also calculated the “overall rate,” which represents the prevalence in the population and is calculated by pooling all studies together (ie, assuming all studies are random samples from the population) and may provide more valid results under heterogeneity. The estimation of average rates is answering the question “What is the mean prevalence seen in studies?,” and the estimation of overall rate estimates the mean prevalence in the population of patients.^30^

If the prevalence of a predefined group was 0% (n=0), we included the study in our meta‐analysis. For the analysis in STATA, 0 was replaced by 0.01 as continuity correction. If the prevalence was 100%, n was replaced by n‐0.01. Studies with an unknown number of participants per predefined group were excluded from the analysis. We estimated heterogeneity with the I^2^ statistic and tau‐squared. STATA 16 was used for all statistical analyses. The significance for the *P* value was set at <0.05.

Because of nonreporting, it was not possible to calculate pooled prevalence of all predefined groups (not possible for dementia, cancer, HF, immunosuppressant condition, treated thyroid disease, kidney failure, chronic obstructive pulmonary disease, mental illness, and atrial fibrillation), of multimorbidity defined as ≥3 chronic conditions, or of polypharmacy.

## RESULTS

### Description of Studies

We included 42 trials, totaling 298 605 patients. Thirty‐two of the trials had been mentioned by the CTTC, of which 28 trials were from the CTTC Collaboration meta‐analysis, 2 trials that were excluded from the CTTC's meta‐analysis because individual patient data were missing, and 2 that were added to the CTTC homepage in or before May 2021. Additionally, we added 10 individual trials from our systematic search. The trials comprised 34 statin trials, 4 trials investigated statin and ezetimibe, 1 only investigated ezetimibe, 2 trials investigated proprotein convertase subtilisin kexin type 9 inhibitors, and 1 trial investigated several lipid‐lowering medications. The setting of 11 trials was primary prevention, and 22 trials focused on secondary prevention. Nine trials included patients in both primary and secondary prevention. All trials specified their inclusion and exclusion criteria for age (>70/>75 years) and sex. See Tables S1 through S3 for the included studies, the baseline characteristics of included studies, and inclusion and exclusion of specific predefined patients' group.

### Risk of Bias of Included Studies

We found that 17 trials met the criteria for low risk of bias in all 5 domains after assessing them with the Cochrane RoB2 tool. Sixteen trials were at moderate risk. Nine trials scored at high risk of bias, including 3 trials for which 2 or 3 moderate risks accumulated (Figure S2).

### Synthesis of Results

**Figure 1:**
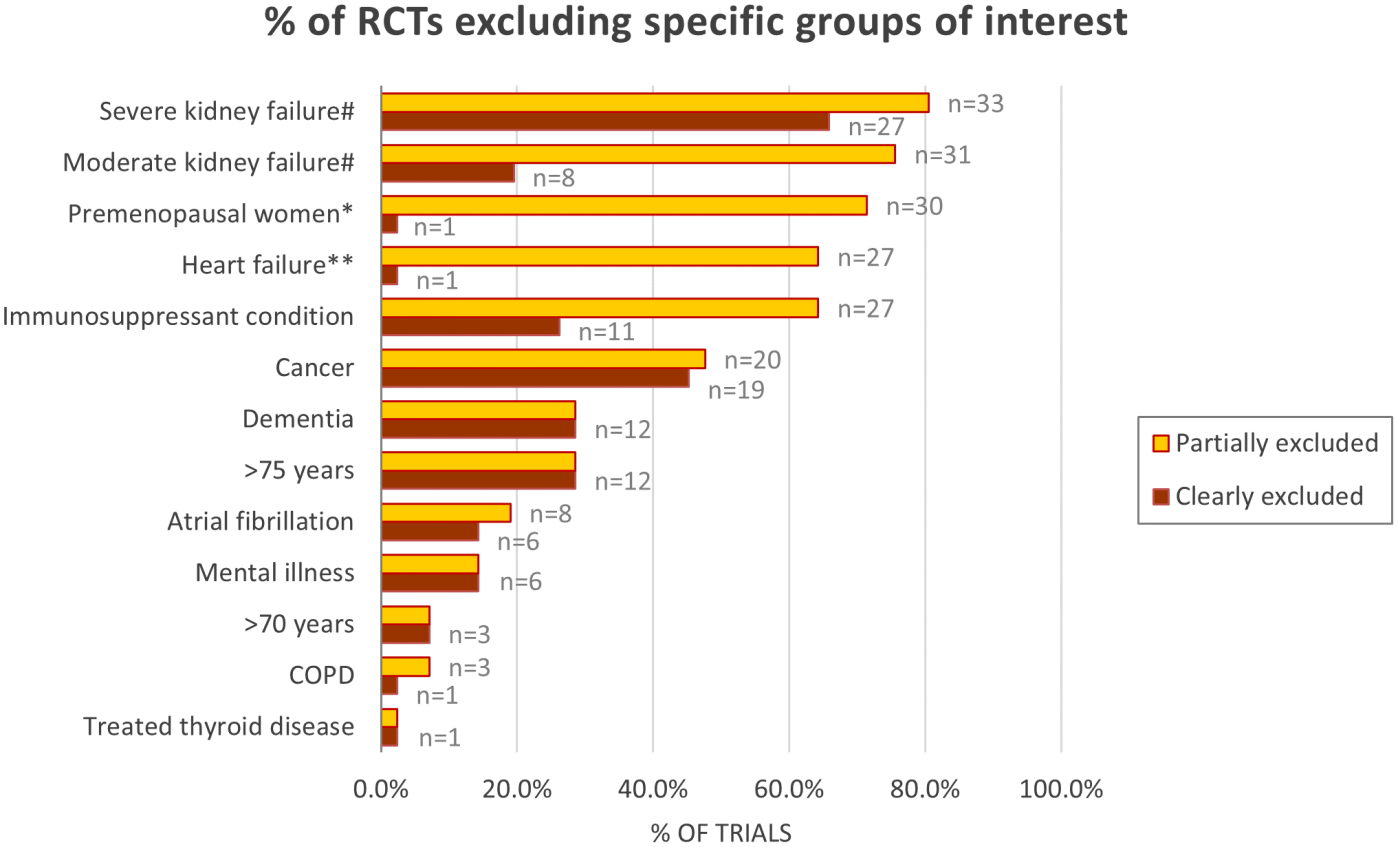
Percentage of randomized controlled trials (n=42, except for kidney failure, n=41) excluding specific predefined groups of interest. Partially excluded: Part of group excluded (eg, not any heart failure but only patients with specific New York Heart Association stages excluded). Clearly excluded: whole group defined as exclusion criterion. No trial specifically defined multimorbidity, polypharmacy, or non‐Whites as an exclusion criterion (0.0%; not shown in the graph). #Kidney failure: n=1 trial excluded for analysis (all patients on hemodialysis). *Women: Group of childbearing potential, pregnant, or lactating women excluded. n=1 trial excluded all women; **See Table S4. COPD indicates chronic obstructive pulmonary disease; and RCTs, randomized controlled trials.

The percentages of studies excluding specific patients' groups are presented in Figure 1 with additional data in Table S4, stratified as “partially excluded” and “clearly excluded.”

Patients with severe CKD were excluded in 65.9% (n=27) of the trials, while 80.5% (n=33) of the trials excluded these patients or part of this group (eg, exclusion criterion: patients with an estimated glomerular filtration rate <20 mL/min or less); 19.5% (n=8) of the trials excluded patients with moderate CKD while 75.6% (n=31) of the trials excluded patients with moderate CKD or part of this group (eg, nephrotic syndrome), and 7.3% (n=3) of the trials did not report whether patients with CKD were excluded or included (Figure S3).

The WOSCOPS (West of Scotland Coronary Prevention Study) trial excluded all women; 71.4% (n=30) of trials excluded groups of women (premenopausal, childbearing potential, pregnant, or lactating women); 21.4% (n=9) trials excluded all premenopausal women, 23.8% (n=10) of trials excluded premenopausal women if no (adequate) contraception, 2.4% (n=1) of trials excluded premenopausal women of childbearing potential, and 28.6% (n=12) of trials excluded pregnant or breastfeeding women.

One trial excluded all patients with HF,^31^ whereas 64.3% (n=27) of trials defined a specific stadium of HF as exclusion criterion (Figure 1 and Table S4). More particularly, 2 trials excluded all patients with HF requiring treatment with digitalis, diuretics, or vasodilators. Two trials excluded all patients with HF New York Heart Association II‐IV, whereas 10 excluded all patients with HF New York Heart Association III‐IV. Seven trials excluded patients with an ejection fraction of <25% or <30%. Four trials excluded patients with decompensated HF. One trial excluded patients with overt HF with unfavorable survival prognosis. One trial excluded patients with “symptomatic” HF or ejection fraction <35%, 1 excluded patients with “severe” HF, and 2 trials excluded patients with systolic HF. Thirty‐one percent of the trials did not report whether patients with HF were excluded or included (Figure S3).

At least 1 group of immunocompromised patients (eg, patients requiring cyclosporine or other treatment with immunosuppressive agents) was excluded from 64.3% (n=27) of trials, while 26.2% (n=11) excluded all patients with serious diseases. Only the ALERT (assessment of Lescol in renal transplantation) trial did not exclude any immunocompromised patients because all patients were transplant recipients. In addition, 26.2% (n=11) of the trials did not report whether they included or excluded immunocompromised patients (Figure S3).

Patients with cancer, including subgroups (eg, history of cancer other than nonmelanoma skin cancer), were excluded in 47.6% (n=20) of trials. Also, 16.7% (n=7) of the trials did not report whether they included or excluded patients with cancer (Figure S3).

Patients with dementia were specifically excluded in 28.6% (n=12) of trials, but 50% of trials circumscribed this exclusion, for example, limiting it to patients who could provide informed consent; 47.6% (n=20) of trials did not state whether they included or excluded patients with dementia (Figure S3).

**Figure 2:**
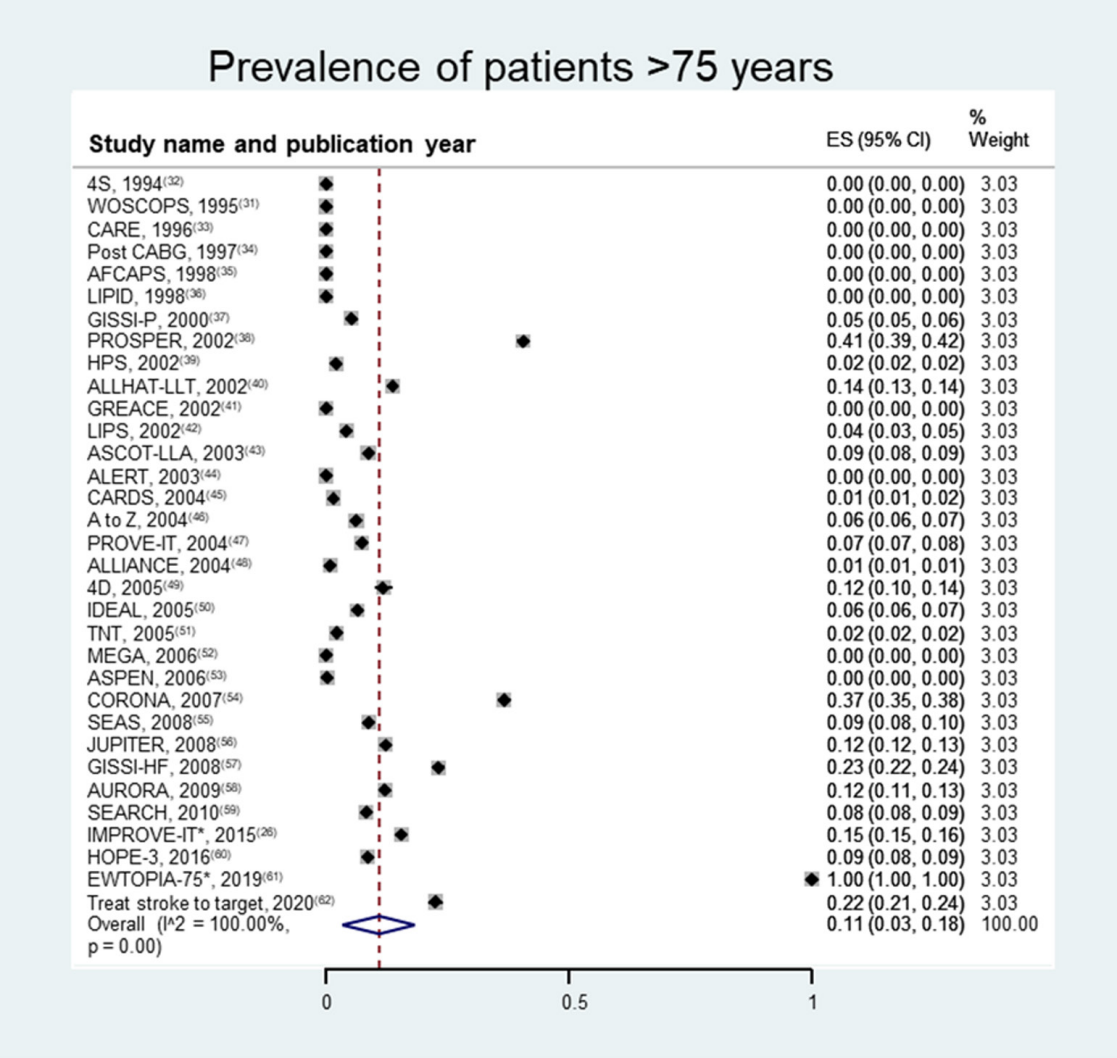
Pooled prevalence of patients >75 years of age. Trials with unknown number of patients excluded: *only data for patients ≥75 years of age are available. Pooled prevalence for patients >75 years of age was 11% (95% CI, 3%–18%). Except for the PROSPER,^38^ CORONA,^54^ and EWTOPIA‐75^61^ trials, the prevalence of patients >75 years of age was <25%. Ten of 33 trials did not include patients >75 years of age. Only the EWTOPIA‐75^61^ trial included only those >75 years of age. ES indicates effect size.

Patients ≥75 years of age were excluded in 28.6% (n=12) of trials; 7.1% (n=3) excluded those >70 years of age. All trials provided information on age (Figure 2).

### Other Groups

Patients with atrial fibrillation, including subgroups of atrial fibrillation such as “uncontrolled,” were excluded from 19% (n=8) of trials; 50% (n=21) did not report whether they included or excluded patients with atrial fibrillation. Patients with chronic obstructive pulmonary disease were excluded from 7.1% (n=3) of trials; 83% (n=35) did not report whether they included or excluded patients with chronic obstructive pulmonary disease. Patients with mental illness were excluded from 14.3% (n=6) of trials; 26.2% excluded patients with mental illness circumscribed (eg, as a “condition that would interfere with optimal participation”); and 73.8% (n=31) of trials did not state whether they included or excluded patients with mental illness. Patients with treated thyroid disease were excluded from 2.4% (n=1) of trials. Sixty percent (n=25) of trials did not report whether they included or excluded patients with treated thyroid disease. No trial mentioned non‐Whites, polypharmacy, or multimorbidity as an exclusion criterion. Because of nonreporting or selective reporting of baseline medications or baseline comorbidities, we could not assess the inclusion or exclusion of patients with polypharmacy in 90.5% (n=38) of trials and with multimorbidity in 11.9% (n=5) of trials (Figure 1; Figure S3).

### Prevalence of Specific Conditions

Pooled prevalence (average of rates) was 25% (95% CI, 0%–49%) for patients >70 years of age and 11% (95% CI, 3%–18%) for patients >75 years of age (Figure 2,^26^, ^31^, ^32^, ^33^, ^34^, ^35^, ^36^, ^37^, ^38^, ^39^, ^40^, ^41^, ^42^, ^43^, ^44^, ^45^, ^46^, ^47^, ^48^, ^49^, ^50^, ^51^, ^52^, ^53^, ^54^, ^55^, ^56^, ^57^, ^58^, ^59^, ^60^, ^61^, ^62^ Figure S4).

**Figure 3:**
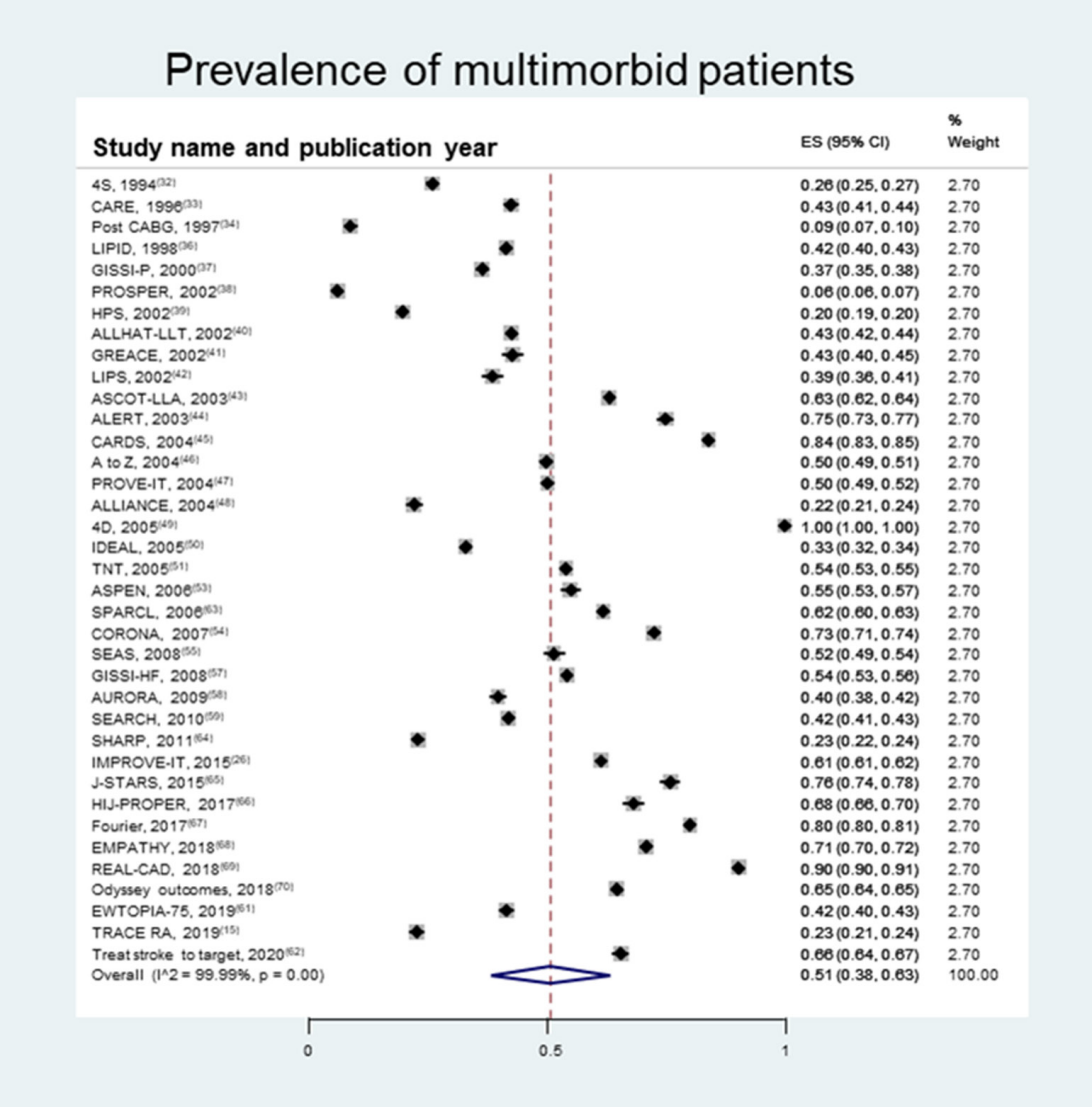
Pooled prevalence of multimorbid patients (≥2 chronic diseases). Trials with unknown number of patients excluded: Pooled prevalence of multimorbidity was at least 51% (95% CI, 38%–63%). The prevalence of multimorbidity varies widely among studies, with only 1 study (4D)^49^ only including multimorbid patients. ES indicates effect size.

Pooled prevalence (average of rates) of multimorbidity was at least 51% (95% CI, 38%–63%) (Figure 3).^15^, ^26^, ^32^, ^33^, ^34^, ^36^, ^37^, ^38^, ^39^, ^40^, ^41^, ^42^, ^43^, ^44^, ^45^, ^46^, ^47^, ^48^, ^49^, ^50^, ^51^, ^53^, ^54^, ^55^, ^57^, ^58^, ^59^, ^61^, ^62^, ^63^, ^64^, ^65^, ^66^, ^67^, ^68^, ^69^, ^70^ Pooled prevalence (average of rates) of women was 30% (95% CI, 24%–37%). Pooled prevalence (average of rates) of non‐Whites was at least 16% (95% CI, 10%–23%).

The overall rates for multimorbidity, participants >70 years of age, participants >75 years of age, women, and non‐Whites were similar to the average rates (Table S5), indicating robust results, despite high heterogeneity.

## DISCUSSION

Patients with conditions such as severe renal dysfunction (creatinine clearance <30 mL/min), severe congestive HF, immunosuppressant conditions, dementia, and mental illness were often excluded in large lipid‐lowering therapy (LLT) trials. No trial, even recent trials, listed multimorbidity or polypharmacy as an exclusion or inclusion criterion, with a pooled prevalence of 51% (95% CI, 38%–63%) of multimorbid patients (defined as ≥2 chronic diseases). One‐third of the trials excluded patients >75 years of age, with a pooled prevalence of elderly patients (>75 years of age) of only 11%.

We specifically assessed the presence of chronic conditions based on data from the baseline tables and found that chronic conditions were often reported in large LLT trials. This contrasts with Jadad et al, who examined 284 trial reports from a variety of medical domains published before 2011 and found that few trials considered multiple chronic diseases.^12^ Jadad et al judged the trials as “not considering chronic diseases” if specific conditions were not named through the article or terms such as comorbidities or coexisting diseases were not mentioned, but we found that the wording “comorbidities or coexisting diseases” was uncommonly used, while this study did not consider how many concomitant diseases the patients had. Using the definition of multimorbidity of being >2 chronic conditions, we found that most of the trials included patients with at least 2 chronic conditions. This proportion is probably lower than patients treated in clinical practice (up to 80% reported).^71^ In accordance with our results, Hanlon et al examined individual participant data from 116 industry‐sponsored trials (122 969 participants) of novel drug treatments for 22 common index conditions and assessed the same comorbidities for the same index conditions in data from a nationally representative community sample of 2.3 million people. Hanlon et al found that >30% of trial participants had ≥2 comorbidities for half of the index conditions.^11^

For several common medical conditions, evidence on statin benefits and side effects is low, because patients with specific common conditions such as severe CKD, severe HF, dementia, and mental illness, or even groups of women were commonly excluded. It is not clear why these medical groups were excluded from these trials, and as previously published usually no reason is given for most of the exclusion criteria.^72^ In addition, in most of the excluded medical conditions lipid‐lowering drugs are not contraindicated. The reason for excluding common conditions may be to avoid situations that could limit the effectiveness of the medications tested or increase the risk of side effects. As to CKD, most of the lipid‐lowering drugs are not contraindicated. The official guidelines differ for the different drugs. For example, ezetimibe does not need an adjustment in severe renal failure, whereas with most statins it is recommended to start at a low dose and to up‐titrate.^73^ Not only are most of the lipid‐lowering drugs not contraindicated in CKD, but it is also essential to include this patient group in RCTs about LLT, because there is an association between decreasing eGFR and CVD risk (independent risk factor)^74^, ^75^, ^76^ but inconclusive evidence on the benefits of statins for advanced CKD.^49^, ^58^, ^77^, ^78^ Because many studies excluded patients with higher‐grade renal insufficiency, it is not possible to assess whether the side effects of statin therapy (eg, interactions with other drugs) or the benefits outweigh the risks. However, in the 4D (Die Deutsche Diabetes Dialyse Studie) trial comparing atorvastatin versus placebo in patients treated with hemodialysis, there was no statistically significant effect on the composite end point of cardiovascular death, myocardial infarction, and stroke.^49^ It may be reasonable not to start statin therapy in multimorbid older patients requiring dialysis or in patients with severe renal insufficiency in the primary prevention,^3^, ^79^ and perhaps it would be reasonable as well to stop statins among those patients, as it is currently explored among multimorbid older adults in primary prevention in a randomized trial.^80^

Regarding chronic HF, the European Society of Cardiology guidelines do not recommend routine administration of statins in patients with HF without other indications for statin use. They do not recommend the discontinuation of statin treatment after the occurrence of HF because there is no evidence of harm.^81^ In contrast, Massumeh et al hypothesized in their review that high doses of statins in patients with long‐term HF might lead to a progression by inhibiting coenzyme Q10 synthesis and intensifying hypertrophy.^82^ Given the exclusion of patients with moderate to severe HF in >60% of all LLT trials and the possibility of worsening HF because of statin therapy, the discontinuation of statins in HF in the primary prevention should be further studied.^80^

Concerning the age of patients, international guidelines offer only cautious treatment recommendations regarding statin initiation in adults >75 years of age.^19^ In accordance with this, we found that the pooled prevalence of elderly patients (>75 years of age) was only 11%, whereas one‐third of trials excluded older adults, suggesting we need more trials that specifically target this population.^17^, ^20^ This is in keeping with the European Society of Cardiology guidelines on dyslipidemia prevention, which give a level of evidence IIb in treating older adults for primary prevention.^81^, ^83^

The European Society of Cardiology guidelines on dyslipidemia prevention mention that only the LIPID (long‐term intervention with pravastatin in ischemic disease) trial has reported significant cardiovascular benefits in the subgroup of women, because women have not been adequately represented in other statin trials.^81^, ^84^ This correlates with our findings that 71.4% of all studies excluded groups of women. The CTTC meta‐analysis of 2015 found that only 27% of all participants were women and that women were generally at lower cardiovascular risk than men included in these trials.^85^ The underrepresentation of women in CVD trials translates into limited knowledge of the efficacy and safety of statins in women compared with men. This is especially true because evidence suggests that women are more likely to discontinue therapies and withdrew consents in large CVD trials.^86^ Furthermore, given the large proportion of female patients who have the occurrence of CVD in later age, the additional exclusion of older adults >75 or 80 years of age can only accentuate the underrepresentativeness of women in clinical trials. Compared with men, women with CVD are underdiagnosed and undertreated.^87^, ^88^ We suggest that future trials should only exclude pregnant women or women wishing to become pregnant and that women with adequate contraception or after menopause should be included in all future lipid‐lowering trials. Our findings have methodological implications for future research and clinical practice. Excluding patients with specific common medical conditions, older adults, or women could be particularly relevant in terms of adverse effects, because specific diseases or characteristics could be associated with higher prevalence of myalgia, for example.^89^ An earlier retrospective study by Hervas Angulo et al determined the characteristics between participants included in large primary prevention trials of hypercholesterolemia and a population of 11 500 inhabitants of Northern Spain with hypercholesterolemia.^16^ They found that between 54% and 97% of participants have been excluded from these cardiovascular trials. In addition, previous studies have shown that very often the exclusion of participants from clinical trials is not sufficiently justified,^72^, ^90^ which our study findings also support. Future trials need to minimize exclusion criteria, specifically when it comes to older adults, women, as well as patients with common medical conditions, and should proactively promote the inclusion of these groups. Considering societal aging and widespread multimorbidity, a better description of the prevalence of multimorbid and participants with polypharmacy is crucial. In addition, subgroup analyses using multivariable models on the effectiveness and possible harms of interventions in this vulnerable population are important, considering pharmacokinetic interactions but also in order to increase adherence and adoption of interventions in the wider population.^91^

Our study had several limitations. First, our pooled prevalence for multimorbidity (defined as >2 chronic conditions) is most likely an underestimate. We had to calculate multimorbidity by proxy, based on data from the baseline tables, and because of missing data we could not calculate multimorbidity in 11.9% of trials. Second, we could not assess polypharmacy in 90% of the trials because of missing data. Finally, we included some studies with high risk of bias. However, for the prevalence of comorbidity and of other conditions, the risk of bias is unlikely to have an impact on the results.

## CONCLUSIONS

Most lipid‐lowering trials excluded large groups of patients managed in clinical practice. Over half of the trials excluded patients with moderate to severe CKD, moderate to severe HF, or with an immunosuppressant condition, which could possibly lead to biased outcomes and possibly more side effects in these groups. Additionally, more than two‐thirds of all studies excluded groups of women, which results in limited knowledge of the efficacy and safety of LLT in women compared with men. One‐third of all studies excluded older adults, and the prevalence of patients >75 years of age over all studies was only 11%. Multimorbid patients (≥2 conditions) represented at least 51% of the included population. Nevertheless, no study specifically reported the inclusion or exclusion of multimorbid patients or patients with polypharmacy. Given that multimorbidity and polypharmacy are common and can contribute to adverse events in drug trials, future studies should minimize those inadequately justified exclusion criteria, promote diversity in the recruitment strategies, and improve equity in cardiovascular research to warrant a generalizable treatment effect estimation and safety for clinical practice.

## Sources of Funding

This study was partly supported by a grant from the Swiss National Science Foundation to study the usefulness of statins among older adults in primary prevention (IICT 33IC30‐193 052 to N.R.). The sponsor played no role in the design, analysis, or reporting of the trial.

## Disclosures

None.

## Supporting information

Data S1
Table S1–S5
Figure S1–S4
