# Supplementary material for: Lipid‐Lowering Trials Are Not Representative of Patients Managed in Clinical Practice: A Systematic Review and Meta‐Analysis of Exclusion Criteria
Source: J Am Heart Assoc. 2022 Dec 24;12(1):e026551. doi: 10.1161/JAHA.122.026551 (PMC9973576; doi:10.1161/JAHA.122.026551)
Supplement: Supplementary file 1 — Data S1 Table S1–S5 Figure S1–S4 [file JAH3-12-e026551-s001.pdf]

Supplemental Material

Supplemental Methods

Data S1. Search strategy.

Date last search **25 May 2021**

	Before deduplication	After deduplication
MEDLINE (Ovid)	1134	1131
EMBASE (Ovid)	2560	1678
Cochrane CENTRAL	2102	648
**Total**	**5796**	**3457**

2339 duplicate records were removed

Ovid MEDLINE(R) and Epub Ahead of Print, In-Process, In-Data-Review & Other Non-Indexed Citations, Daily and Versions(R) <1946 to May 24, 2021>

1 exp cholesterol/
2 (cholesterol* or lipid* or LDL).ab,ti,kf.
3 1 or 2
4 exp Anticholesteremic Agents/
5 ((inhibit* adj3 ("hmg-coa*" or "Hydroxymethylglutaryl CoA*" or "Hydroxymethylglutaryl-Coenzyme A")) or statins or statin or simvastatin or rosuvastatin or pravastatin or pitavastatin or mevastatin or lovastatin or glenvastatin or fluvastatin or fluindostatin or dalvastatin or crilvastatin or atorvastatin or cerivastatin or bervastatin or medostatin).ab,kf,ti,nm.
6 (altoprev or altocor or baycol or canef or cranoc or compactin or crestor or lescol or lipitor or lipex or lipostat or livalo or locol or lochol or mevinolin or mevacor or mevalotin or mevinacor or monacolin or pravachol or pitava or pravachol or pravasin or zocor).mp.
7 (antichol* or antihyperchol* or hypochol* or hypolipidemic* or antihyperlipidemic* or anti-hyperlipidemic* or Ezetimibe or PCSK9 inhibitor* or Alirocumab or evolocumab or non-statin*).ab,ti,kf.
8 4 or 5 or 6 or 7
9 exp cardiovascular diseases/ or exp mortality/
10 (((cardiovascular or heart or coronar* or cardiac) adj3 (disease* or event* or attack* or mortalit* or death* or arrest*)) or cvd or cvds or CV-mortalit* or MACE or angina or ((heart or cardia* or myocard*) adj3 (ischemi* or ischaemi* or fail* or insufficien*)) or ((myocard* or heart or cardiac) adj3 (infarct* or attack*)) or (cerebrovascular* adj3 (accident* or event*)) or cva or stroke* or ((brain or cerebral) adj3 (ischemi* or ischaemi*))).ti,ab.
11 9 or 10
12 3 and 8 and 11
13 randomized controlled trial.pt.
14 (random$ or placebo$ or single blind$ or double blind$ or triple blind$).ti,ab.
15 (retraction of publication or retracted publication).pt.
16 or/13-15
17 (animals not humans).sh.
18 ((comment or editorial or meta-analysis or practice-guideline or review or letter) not randomized controlled trial).pt. 4822254
19 (random sampl$ or random digit$ or random effect$ or random survey or random regression).ti,ab. not randomized controlled trial.pt
20 16 not (17 or 18 or 19)
21 12 and 20
22 limit 21 to yr="2015 -Current"

mp=(multi-purpose field): title, abstract, original title, name of substance word, subject heading word, floating sub-heading word, author keyword heading word, organism supplementary concept word, protocol supplementary concept word, rare disease supplementary concept word, unique identifier, synonyms

nm=Name of substance word
kf=Author keyword heading word

BMJ Best Practice Study design search filters
Medline randomised controlled trial strategy
https://bestpractice.bmj.com/info/toolkit/learn-ebm/study-design-search-filters/

Embase (Ovid) <1974 to 2021 May 24>

1 cholesterol/
2 (cholesterol* or lipid* or LDL).ti,ab,kw.
3 1 or 2
4 exp hypocholesterolemic agent/
5 ((inhibit* adj3 ("hmg-coa*" or "Hydroxymethylglutaryl CoA*" or "Hydroxymethylglutaryl-Coenzyme A")) or statins or statin or simvastatin or rosuvastatin or pravastatin or pitavastatin or mevastatin or lovastatin or glenvastatin or fluvastatin or fluindostatin or dalvastatin or crilvastatin or atorvastatin or cerivastatin or bervastatin or medostatin).ab,kw,ti,rn.
6 (altoprev or altocor or baycol or canef or cranoc or compactin or crestor or lescol or lipitor or lipex or lipostat or livalo or locol or lochol or mevinolin or mevacor or mevalotin or mevinacor or monacolin or pravachol or pitava or pravachol or pravasin or zocor).mp.
7 (antichol* or antihyperchol* or hypochol* or hypolipidemic* or antihyperlipidemic* or anti-hyperlipidemic* or Ezetimibe or PCSK9 inhibitor* or Alirocumab or evolocumab or non-statin*).ti,ab,kw.
8 4 or 5 or 6 or 7
9 exp cardiovascular disease/ or exp mortality/
10 (((cardiovascular or heart or coronar* or cardiac) adj3 (disease* or event* or attack* or mortalit* or death* or arrest*)) or cvd or cvds or CV-mortalit* or MACE or angina or ((heart or cardia* or myocard*) adj3 (ischemi* or ischaemi* or fail* or insufficien*)) or ((myocard* or heart or cardiac) adj3 (infarct* or attack*)) or (cerebrovascular* adj3 (accident* or event*)) or cva or stroke* or ((brain or cerebral) adj3 (ischemi* or ischaemi*))).ti,ab.
11 9 or 10
12 3 and 8 and 11
13 (random$ or placebo$ or single blind$ or double blind$ or triple blind$).ti,ab.
14 RETRACTED ARTICLE/
15 13 or 14
16 (animal$ not human$).sh,hw.
17 (book or conference paper or editorial or letter or review).pt. not exp randomized controlled trial/
18 (random sampl$ or random digit$ or random effect$ or random survey or random regression).ti,ab. not exp randomized controlled trial/
19 15 not (16 or 17 or 18)
20 12 and 19
21 limit 20 to yr="2015 -Current"

mp=(multi-purpose field) title, abstract, heading word, drug trade name, original title, drug manufacturer, author keyword, floating subheading word, candidate term word

rn=CAS Registry Number (chemical names)
kw=Author keyword

BMJ Best Practice Study design search filters
Embase randomised controlled trial strategy
https://bestpractice.bmj.com/info/toolkit/learn-ebm/study-design-search-filters/

Cochrane CENTRAL
Cochrane Central Register of Controlled **Trials**

#1 (cholesterol* or lipid* or LDL):ti,ab,kw
#2 (statin or statins or (inhibit* NEAR/3 ("hmg coa" or "Hydroxymethylglutaryl CoA" or "Hydroxymethylglutaryl Coenzyme")) or atorvastatin or cerivastatin or crilvastatin or dalvastatin or fluindostatin or fluvastatin or glenvastatin or lovastatin or mevastatin or pitavastatin or pravastatin or rosuvastatin or simvastatin)
#3 (altoprev or altocor or baycol or canef or cranoc or compactin or crestor or lescol or lipitor or lipex or lipostat or livalo or locol or lochol or mevinolin or mevacor or mevalotin or mevinacor or monacolin or pravachol or pitava or pravachol or pravasin or zocor)
#4 (antichol* or antihyperchol* or hypochol* or hypolipidemic* or antihyperlipidemic* or anti-hyperlipidemic* or Ezetimibe or PCSK9 inhibitor* or Alirocumab or evolocumab or (non NEXT statin*))
#5 #2 or #3 or #4
#6 (((cardiovascular or heart or coronar* or cardiac) NEAR/3 (disease* or event* or attack* or mortalit* or death* or arrest*)) or cvd or cvds or CV-mortalit* or MACE or angina or ((heart or cardia* or myocard*) NEAR/3 (ischemi* or ischaemi* or fail* or insufficien*)) or ((myocard* or heart or cardiac) NEAR/3 (infarct* or attack*)) or (cerebrovascular* NEAR/3 (accident* or event*)) or cva or stroke* or ((brain or cerebral) NEAR/3 (ischemi* or ischaemi*)))
#7 #1 AND #5 AND #6
#8 Filter year range: 2015 to 2021

Table S1: Abbreviations of study titles

Abbreviations	Study title
SSSS (4S)	The Scandinavian Simvastatin Survival Study<br>*Randomised trial of cholesterol lowering in 4444 patients with coronary heart disease: the Scandinavian Simvastatin Survival Study*
WOSCOPS	The West of Scotland Coronary Prevention Study<br>*Prevention of coronary heart disease with pravastatin in men with hypercholesterolemia*
CARE	The Cholesterol and Recurrent Events Trial<br>*The effect of Pravastatin on coronary events after myocardial infarction in patients with average cholesterol levels*
AFCAPS/TexCaps	Air Force Texas Coronary Atherosclerosis Prevention Study<br>*Primary prevention of acute coronary events with Lovastatin in men and women with average cholesterol levels*
LIPID	The Long-Term Intervention with Pravastatin in Ischaemic Disease Study<br>*Prevention of cardiovascular events and death with pravastatin in patients with coronary heart disease and a broad range of initial cholesterol levels*
GISSI-P	Gruppo Italiano per lo Studio della Sopravvivenza nell'Infarto Miocardico Prevenzione trial<br>*Results of the low-dose (20mg) pravastatin GISSI Prevenzione trial in 4271 patients with recent myocardial infarction: do stopped trials contribute to overall knowledge?*
HPS	The Heart Protection Study<br>*Heart Protection Study of cholesterol lowering with simvastatin in 20 536 high-risk individuals: a randomised placebo-controlled trial*
LIPS	The Lescol Intervention Prevention Study

	Fluvastatin for Prevention of Cardiac Events Following Successful First Percutaneous Coronary Intervention
PROSPER	The PROspective Study of Pravastatin in the Elderly at Risk<br>*Pravastatin in elderly individuals at risk of vascular disease: a randomised controlled trial*
ALERT	Assessment of LEscol in Renal Transplantation Study<br>*Effect of fluvastatin on cardiac outcomes in renal transplant recipients: a multicenter, randomized, placebo-controlled trial*
ASCOT-LLA	The Anglo-Scandinavian Cardiac Outcomes Trial--Lipid Lowering Arm<br>*Prevention of coronary and stroke events with atorvastatin in hypertensive patients who have average or lower-than-average cholesterol concentrations, in the Anglo-Scandinavian Cardiac Outcomes Trial-Lipid Lowering Arm: a multicenter randomised controlled trial*
4D	Die Deutsche Diabetes Dialyse Studie<br>*Atorvastatin in Patients with Type 2 Diabetes Mellitus undergoing hemodialysis*
CARDS	Collaborative Atorvastatin Diabetes Study<br>*Primary prevention of cardiovascular disease with atorvastatin in type 2 diabetes in the Collaborative Atorvastatin Diabetes Study: multicenter randomised placebo-controlled trial*
ASPEN	The Atorvastatin Study for Prevention of Coronary Heart Disease Endpoints in Non-Insulin-Dependent Diabetes Mellitus<br>*Efficacy and Safety of Atorvastatin in the Prevention of Cardiovascular End Points in Subjects with Type 2 Diabetes*
SPARCL	The Stroke Prevention by Aggressive Reduction in Cholesterol Levels trial<br>*High-Dose Atorvastatin after Stroke or Transient Ischemic Attack*
CORONA	Controlled Rosuvastatin multinational study in heart failure<br>*Rosuvastatin in Older Patients with Systolic Heart Failure*
GISSI-HF	Gruppo Italiano per lo Studio della Sopravvivenza nell'Insufficienza Cardiaca trial

	Effect of Rosuvasttin in patients with chronic heart failure: a randomised, double-blind, placebo-controlled trial
	Justification for the Use of Statins in Prevention: an Intervention Trial Evaluating Rosuvastatin
JUPITER	*Rosuvastatin to prevent vascular events in men and women with elevated C-Reactive Protein*
	A Study to Evaluate the Use of Rosuvastatin in Subjects on Regular Hemodialysis:
AURORA	*An Assessment of Survival and Cardiovascular Events*
	Rosuvastatin and Cardiovascular Events in patients undergoing hemodialysis
	Heart Outcomes Prevention Evaluation-3 trial
HOPE-3	*Cholesterol Lowering in Intermediate-Risk Persons without Cardiovascular Disease*
	Study of Heart and Renal Protection
SHARP	*The effects of lowering LDL cholesterol with simvastatin plus ezetimibe in patients with chronic kidney disease: a randomised placebo-controlled trial*
	The Simvastatin and Ezetimibe in Aortic Stenosis trial
SEAS	*Intensive Lipid Lowering with Simvastatin and Ezetimibe in Aortic Stenosis*
	Improved Reduction of Outcomes: Vytorin Efficacy International Trial
IMPROVE-IT	*Ezetimibe added to statin therapy after acute coronary syndromes*
	Further Cardiovascular Outcomes Research With PCSK9 Inhibition in Subjects With
FOURIER	Elevated Risk trial
	Evolocumab and Clinical outcomes in Patients with cardiovascular disease
	Evaluation of Cardiovascular Outcomes After an Acute Coronary Syndrome During
Odyssey outcomes	Treatment With Alirocumab trial
	Alirocumab and Cardiovascular Outcomes after acute coronary syndrome
	The Japan Statin Treatment Against Recurrent Stroke Study
J-STARS	*The Japan Statin Treatment Against Recurrent Stroke: A multicenter, randomized, open-label, parallel-group study*
ALLIANCE	The Aggressive Lipid-Lowering Initiation Abates New Cardiac Events study

	Clinical Outcomes in managed-care patients with coronary heart disease treated aggressively in lipid-lowering disease management clinics: The Alliance study
MEGA	The Management of Elevated Cholesterol in the Primary Prevention Group of Adult Japanese Study<br>*Primary Prevention of cardiovascular disease with pravastatin in Japan: a prospective randomised controlled trial*
EWTOPIA 75	Ezetimibe Lipid-Lowering Trial on Prevention of Atherosclerotic Cardiovascular Disease in 75 or Older
ALLHAT-LLT	Antihypertensive and Lipid-Lowering Treatment to Prevent Heart Attack Trial<br>*Major outcomes in moderately Hypercholesterolemic, Hypertensive Patients Randomized to Pravastatin vs Usual Care*
GREACE	The Greek Atorvastatin and Coronary Heart Disease Evaluation Study<br>*Treatment with Atorvastatin to the National Cholesterol Educational Program Goal Versus "Usual" Care in Secondary Coronary Heart Disease Prevention*
EMPATHY	The standard versus intEnsive statin therapy for hypercholesteroleMic Patients with diAbetic retinopaTHY study<br>*Intensive Treat-to-Target Statin Therapy in High-Risk Japanese patients with Hypercholesterolemia and Diabetic Retinopathy: Report of a Randomized Study*
PROVE-IT	The Pravastatin or Atorvastatin Evaluation and Infection Therapy trial<br>*Intensive versus moderate lipid lowering with statins after acute coronary syndromes*
Post CABG	The post coronary artery bypass graft trial<br>*The effect of aggressive lowering of low-density lipoprotein cholesterol levels and low-dose anticoagulation on obstructive changes in saphenous-vein coronary-artery bypass grafts*
TNT	Treating to New Targets Study<br>*Intensive Lipid Lowering with Atorvastatin in Patients with Stable Coronary Disease*
IDEAL	The Incremental Decrease in End Points Through Aggressive Lipid Lowering Study

	High-dose Atorvastatin vs usual-dose Simvastatin for secondary prevention after myocardial infarction: the IDEAL study: a randomized controlled trial
SEARCH	Study of the effectiveness of additional reductions in cholesterol and homocysteine<br>*Intensive lowering of LDL cholesterol with 80mg versus 20mg simvastatin daily in 12064 survivors of myocardial infarction: a double-blind randomised trial*
A to Z	Aggrastat to Zocor trial<br>*Early intensive vs a delayed conservative simvastatin strategy in patients with acute coronary syndromes*
TST	Treat stroke to target trial<br>*A Comparison of Two LDL Cholesterol Targets after Ischemic Stroke*
HIJ-PROPER	Heart Institute of Japan-PRoper level of lipid lOwering with Pitavastatin and Ezetimibe in acute coRonary syndrome<br>*Low-density lipoprotein cholesterol targeting with pitavastatin + ezetimibe for patients with acute coronary syndrome and dyslipidaemia: the HIJ-PROPER study, a prospective, open-label, randomized trial*
REAL-CAD	Randomized Evaluation of Aggressive or Moderate Lipid Lowering Therapy With Pitavastatin in Coronary Artery Disease<br>*High-Dose versus Low-Dose Pitavastatin in Japanese Patients with stable coronary artery disease*
TRACE RA	TRial of Atorvastatin for the primary prevention of Cardiovascular Events in Rheumatoid Arthritis<br>*A Multicenter, randomized, placebo-controlled trial of Atorvastatin for the primary prevention of cardiovascular events in patients with Rheumatoid Arthritis*

Table S2: Inclusion and exclusion of specific predefined patients' group

Study name \ Patient's group	>70 years	>75 years	Multimorbidity (≥2 conditions)	Polypharmacy (≥5 drugs)	Women	Non-white	Dementia	Active Cancer	Heart failure	Immuno-suppressant condition	Treated for thyroid disease	Severe kidney failure	Moderate kidney failure	COPD	Mental illness	Atrial fibrillation
**4S**	Excl	Excl	Incl	Unknown	Partially	Unknown	Excl	Probably	Partially	Probably	Unknown	Probably	Probably	Probably	Probably	Excl
**WOSCOPS**	Excl	Excl	Unknown	Unknown	Excl	Unknown	Excl	Probably	Excl	Probably	Unknown	Excl	Excl	Excl	Excl	Excl
**CARE**	Incl	Excl	Incl	Unknown	Partially	Incl	Excl	Excl	Partially	Excl	Incl	Excl	Excl	Unknown	Excl	Unknown
**Post CABG**	Incl	Excl	Incl	Unknown	Incl	Incl	Unknown	Unknown	Partially	Unknown	Unknown	Unknown	Unknown	Unknown	Unknown	Unknown
**AFCAPS**	Incl	Excl	Unknown	Unknown	Partially	Incl	Probably	Probably	Unknown	Unknown	Incl	Partially	Partially	Unknown	Unknown	Unknown
**LIPID**	Incl	Excl	Incl	Unknown	Incl	Unknown	Unknown	Unknown	Partially	Partially	Incl	Excl	Excl	Unknown	Unknown	Unknown
**GISSI-P**	Incl	Incl	Incl	Unknown	Incl	Unknown	Excl	Excl	Partially	Partially	Unknown	Partially	Partially	Unknown	Excl	Unknown
**ALLHAT-LLT**	Incl	Incl	Incl	Unknown	Incl	Incl	Unknown	Probably	Partially	Excl	Incl	Excl	Partially	Unknown	Unknown	Unknown
**HPS**	Incl	Incl	Incl	Unknown	Partially	Unknown	Excl	Excl	Partially	Partially	Unknown	Excl	Partially	Partially	Excl	Unknown
**LIPS**	Incl	Incl	Incl	Unknown	Partially	Unknown	Excl	Excl	Partially	Unknown	Incl	Excl	Excl	Unknown	Probably	Unknown
**PROSPER**	Incl	Incl	Incl	Unknown	Incl	Unknown	Excl	Excl	Partially	Partially	Incl	Excl	Partially	Unknown	Probably	Excl
**GREACE**	Incl	Excl	Incl	Unknown	Partially	Unknown	Unknown	Probably	Partially	Unknown	Unknown	Excl	Excl	Unknown	Unknown	Unknown
**ALERT**	Incl	Excl	Incl	Incl	Partially	Unknown	Excl	Partially	Unknown	Incl	Unknown	Incl	Incl	Unknown	Probably	Unknown
**ASCOT-LLA**	Incl	Incl	Incl	Unknown	Partially	Incl	Excl	Probably	Partially	Excl	Unknown	Excl	Partially	Unknown	Excl	Partially
**A to Z**	Incl	Incl	Incl	Unknown	Partially	Unknown	Unknown	Unknown	Incl	Partially	Unknown	Excl	Partially	Unknown	Unknown	Partially
**ALLIANCE**	Incl	Incl	Incl	Unknown	Partially	Incl	Unknown	Excl	Partially	Unknown	Unknown	Unknown	Unknown	Unknown	Unknown	Unknown

**CARDS**	Incl	Excl	Incl	Unknown	Incl	Incl	Probably	Probably	Partially	Excl	Incl	Excl	Excl	Unknown	Unknown	Probably
**PROVE-IT**	Incl	Incl	Incl	Unknown	Incl	Incl	Unknown	Probably	Unknown	Partially	Unknown	Excl	Partially	Unknown	Unknown	Unknown
**4D**	Incl	Incl	Incl	Unknown	Partially	Unknown	Unknown	Probably	Partially	Probably	Incl	n.a.	n.a.	Unknown	Unknown	Unknown
**IDEAL**	Incl	Incl	Incl	Unknown	Partially	Incl	Probably	Probably	Partially	Excl	Incl	Partially	Partially	Unknown	Unknown	Incl
**TNT**	Incl	Excl	Incl	Unknown	Partially	Incl	Unknown	Excl	Partially	Excl	Incl	Partially	Partially	Unknown	Unknown	Incl
**ASPEN**	Incl	Excl	Incl	Incl	Partially	Incl	Unknown	Unknown	Partially	Excl	Unknown	Excl	Probably	Unknown	Unknown	Incl
**MEGA**	Excl	Excl	Unknown	Unknown	Partially	Incl	Unknown	Excl	Unknown	Partially	Unknown	Partially	Partially	Unknown	Unknown	Excl
**SPARCL**	Incl	Incl	Incl	Unknown	Partially	Incl	Excl	Unknown	Unknown	Excl	Unknown	Excl	Probably	Unknown	Excl	Excl
**CORONA**	Incl	Incl	Incl	Unknown	Incl	Unknown	Unknown	Excl	Partially	Partially	Incl	Excl	Partially	Unknown	Unknown	Incl
**GISSI-HF**	Incl	Incl	Incl	Unknown	Partially	Unknown	Unknown	Probably	Incl	Unknown	Unknown	Excl	Partially	Incl	Unknown	Incl
**JUPITER**	Incl	Incl	Unknown	Unknown	Partially	Incl	Probably	Excl	Unknown	Excl	Incl	Excl	Partially	Unknown	Unknown	Unknown
**AURORA**	Incl	Incl	Incl	Unknown	Incl	Incl	Unknown	Excl	Unknown	Partially	Incl	Incl	Incl	Unknown	Unknown	Unknown
**SEARCH**	Incl	Incl	Incl	Unknown	Partially	Unknown	Probably	Probably	Unknown	Partially	Unknown	Excl	Partially	Unknown	Unknown	Unknown
**J-STARS**	Incl	Incl	Incl	Unknown	Incl	Incl	Incl	Excl	Unknown	Unknown	Unknown	Excl	Partially	Unknown	Unknown	Unknown
**HOPE-3**	Incl	Incl	Unknown	Unknown	Partially	Incl	Probably	Probably	Unknown	Partially	Unknown	Excl	Excl	Unknown	Unknown	Unknown
**REAL-CAD**	Incl	Incl	Incl	Unknown	Partially	Incl	Probably	Excl	Partially	Partially	Unknown	Incl	Incl	Unknown	Unknown	Incl
**EMPATHY**	Incl	Incl	Incl	Unknown	Partially	Incl	Unknown	Excl	Partially	Unknown	Unknown	Excl	Partially	Unknown	Unknown	Unknown
**TRACE RA**	Incl	Incl	Incl	Unknown	Partially	Incl	Probably	Probably	Partially	Partially	Incl	Excl	Excl	Unknown	Unknown	Probably
**TST**	Incl	Incl	Incl	Unknown	Partially	Incl	Unknown	Excl	Unknown	Partially	Unknown	Unknown	Unknown	Unknown	Unknown	Incl
**SEAS**	Incl	Incl	Incl	Unknown	Incl	Incl	Unknown	Incl	Partially	Unknown	Incl	Excl	Partially	Incl	Unknown	Incl
**SHARP**	Incl	Incl	Incl	Unknown	Partially	Incl	Unknown	Excl	Unknown	Partially	Unknown	Incl	Incl	Partially	Unknown	Unknown

**IMPROVE-IT**	Incl	Incl	Incl	Incl	Partially	Incl	Unknown	Unknown	Partially	Partially	Unknown	Excl	Partially	Unknown	Unknown	Incl
**HIJ-PROPER**	Incl	Incl	Incl	Unknown	Partially	Incl	Probably	Excl	Partially	Excl	Unknown	Excl	Partially	Unknown	Unknown	Incl
**EWTOPIA-75**	Incl	Incl	Incl	Unknown	Incl	Incl	Excl	Excl	Unknown	Unknown	Excl	Excl	Partially	Incl	Probably	Excl
**FOURIER**	Incl	Incl	Incl	Unknown	Partially	Incl	Excl	Excl	Partially	Excl	Incl	Partially	Partially	Unknown	Unknown	Incl
**ODYSSEY OUTCOMES**	Incl	Incl	Incl	Incl	Partially	Incl	Unknown	Unknown	Partially	Unknown	Unknown	Excl	Partially	Unknown	Unknown	Unknown

Excl: Patient group as a clearly defined exclusion criterion. Partially: Part of patients' group excluded (e.g. only patients with heart failure NYHA III-IV excluded).Probably: Patients' group not clearly mentioned; medical condition circumscribed. Unknown: Unknown (no information available; mentioned neither as an inclusion nor as an exclusion criterion, no information in baseline table). n.a.: Kidney failure#: n=1 trial excluded for analysis (all patients on hemodialysis)

Table S3: Baseline characteristics of the included trials

Control arm: Placebo or no treatment								
Study Name	Prevention type	Intervention	Mean age (y)	Men (prevalence %)	Follow-up (y)	Overall N	Overall LLT	Overall Control
4S	Secondary	20mg Simvastatin	58.9	81.4	5.4	4444	2221	2223
WOSCOPS	Primary	40mg Pravastatin	55.2	100	4.9	6595	3302	3293
CARE	Secondary	40mg Pravastatin	59	86	5	4159	2081	2078
AFCAPS/TexCaps	Primary	20-40mg Lovastatin	58	85	5.2	6605	3304	3301

LIPID	Secondary	40mg Pravastatin	62	83	6.1	9014	4512	4502
GISSI-P	Secondary	20mg Pravastatin	59.9	86.3	1.92	4271	2138	2133
HPS	Primary & Secondary	40mg Simvastatin	NR	75.3	5	20536	10269	10267
LIPS	Secondary	80mg Fluvastatin	60	83.8	3.9	1677	844	833
PROSPER	Primary & Secondary	40mg Pravastatin	75.3	48.3	3.2	5804	2891	2913
ALERT	Primary & Secondary	40mg Fluvastatin	49.8	66	5.1	2102	1050	1052
ASCOT-LLA	Primary & Secondary	10mg Atorvastatin	63.1	81.2	3.3	10305	5168	5137
4D	Primary & Secondary	20mg Atorvastatin	65.7	54	3.93	1255	619	636

CARDS	Primary	10mg Atorvastatin	61.7	68	3.9	2838	1428	1410
ASPEN	Primary & Secondary	10mg Atorvastatin	61.1	66.3	4	2410	1211	1199
SPARCL	Secondary	80mg Atorvastatin	62.8	59.7	4.9	4731	2365	2366
CORONA	Secondary	10mg Rosuvastatin	73	76	2.7	5011	2514	2497
GISSI-HF	Primary & Secondary	10mg Rosuvastatin	68	77.4	3.9	4574	2285	2289
JUPITER	Primary	20mg Rosuvastatin	66 (median)	61.8	1.9	17802	8901	8901
AURORA	Primary & Secondary	10mg Rosuvastatin	64.2	62.1	3.2	2773	1389	1384
HOPE-3	Primary	10mg Rosuvastatin	65.7	53.8	5.6	12705	6361	6344

SHARP	Primary	20mg Simvastatin + 10mg Ezetimibe	62	62.6	4.9	9270	4650	4620
SEAS	Primary	40mg Simvastatin + 10mg Ezetimibe	67.6	61.4	4.35	1873	944	929
IMPROVE-IT	Secondary	40mg Simvastatin + 10mg Ezetimibe	63.6	75.7	6	18144	9067	9077
FOURIER	Secondary	Evolocumab 140mg or 420mg	62.5	75.4	2.2	27564	13784	13780
Odyssey outcomes	Secondary	Alirocumab 75mg	58.6	74.8	2.8	18924	9462	9462
J-STARS	Secondary	10mg Pravastatin	66.2	68.8	4.9	1578	793	785
TRACE RA	Primary	40mg Atorvastatin	61	25.8	2.51	3002	1504	1498

Active Control or Usual care

Study Name	Prevention type	Intervention	Control	Mean age (y)	Men prevalence (%)	Follow-up (y)	Overall N	Overall LLT	Overall Control
ALLIANCE	Secondary	10-80mg Atorvastatin	Usual care	61.2	82.2	4.3	2442	1217	1225
MEGA	Primary	10-20mg Pravastatin	Diet (pyhsician could prescribe mild hypo-lipidemic drugs)	58.3	31.6	5.3	7832	3866	3966
EWTOPIA 75	Primary	10mg Ezetimibe	Usual care	80.6	25.6	4.1	3411	1716	1695
ALLHAT-LLT	Primary & Secondary	40mg Pravastatin	Usual care	66.4	51.2	4.8	10355	5170	5185
GREACE	Secondary	10-80mg Atorvastatin	Usual care	58.5	78.5	3	1600	800	800

EMPATHY	Primary	LDL-goal <70mg/dl (statin)	LDL goal 100-120mg/dl (statin)	63.1	47.7	3.1	5042	2518	2524
PROVE-IT	Secondary	80mg Atorvastatin	40mg (-80mg) Pravastatin	58.2	78	2	4162	2099	2063
Post CABG	Secondary	40mg Lovastatin +/- Cholestyramine	2.5mg Lovastatin +/- Cholestyramine	61.5	92	4.3	1351	676	675
TNT	Secondary	80mg Atorvastatin	10mg Atorvastatin	61	81	4.9	10001	4995	5006
IDEAL	Secondary	80mg Atorvastatin	20mg Simvastatin	61.7	80.9	4.8	8888	4439	4449
SEARCH	Secondary	80mg Simvastatin	20mg Simvastatin	64.2	83	6.7	12064	6031	6033

A to Z	Secondary	40 mg Simvastatin for 1 month followed by 80mg thereafter	Placebo for 4 months followed by 20 mg Simvastatin	61 (median)	75.5	1.97	4497	2265	2232
TST	Secondary	LDL <70mg/dl	LDL 90-110mg/dl	66.7	67.6	3.5	2860	1430	1430
HIJ-PROPER	Secondary	Standard-dose Pitavastatin plus ezetimibe (LDL target <70mg/dl)	Pitavastatin (LDL target 90-100mg/dl)	65.6	75.5	3.86	1721	864	857
REAL-CAD	Secondary	4mg Pitavastatin	1mg Pitavastatin	68.1	82.6	3.9	12413	6199	6214

Table S4: Exclusion criteria HF

Exclusion criterion HF	Trials (n)
HF treated with digitalis, diuretics, vasodilators	1
HF treated with digoxin	1
NYHA II-IV	2
NYHA III-IV	7
NYHA III-IV or EF<30%	2
NYHA III-IV persisting despite treatment or EF <25%	1
EF <30%	3
EF <25%	1
Symptomatic HF or EF <35%	1
Severe HF	1
Systolic HF	2
Overt HF (unfavorable survival prognosis)	1
Congestive HF within last 3 months	1
Decompensated congestive HF or need for inotropic therapy (digitalis allowed)	1
Decompensated congestive HF 24h prior to screening	1
Hemodynamic instability 24h before enrolment	1

Table S5: Pooled prevalence - comparison overall and average rates

	Prevalence of Multimorbidity in % (95% CI)	Prevalence of participants >75years in % (95% CI)	Prevalence of participants >70 years in % (95% CI)	Prevalence of women in % (95% CI)	Prevalence of non-whites in % (95% CI)
Average rates	51 (38-63)	11 (3-18)	25 (0-49)	30 (24-37)	16 (10-23)
Overall rates	52.5 (52.3-52.7)	10 (9.9-10.2)	24 (24.0-24.4)	29(28.7-29.0)	21 (20.8-21.2)

*Average rate is calculated from meta-analysis based on the Freeman-Tukey method and estimates the mean of the mean prevalence across studies. It is answering the question "what is the mean prevalence seen across studies"

*Overall rates is calculated by pooling all studies together (i.e. assuming all studies are random samples from the population) and estimates the mean prevalence in the population of patients (ref. Hansen S, Rice K. Exact inference for fixed-effects meta-analysis of proportions. Research Synthesis Methods. 2022;13(2):204-13)

Figure S1: Study flow chart

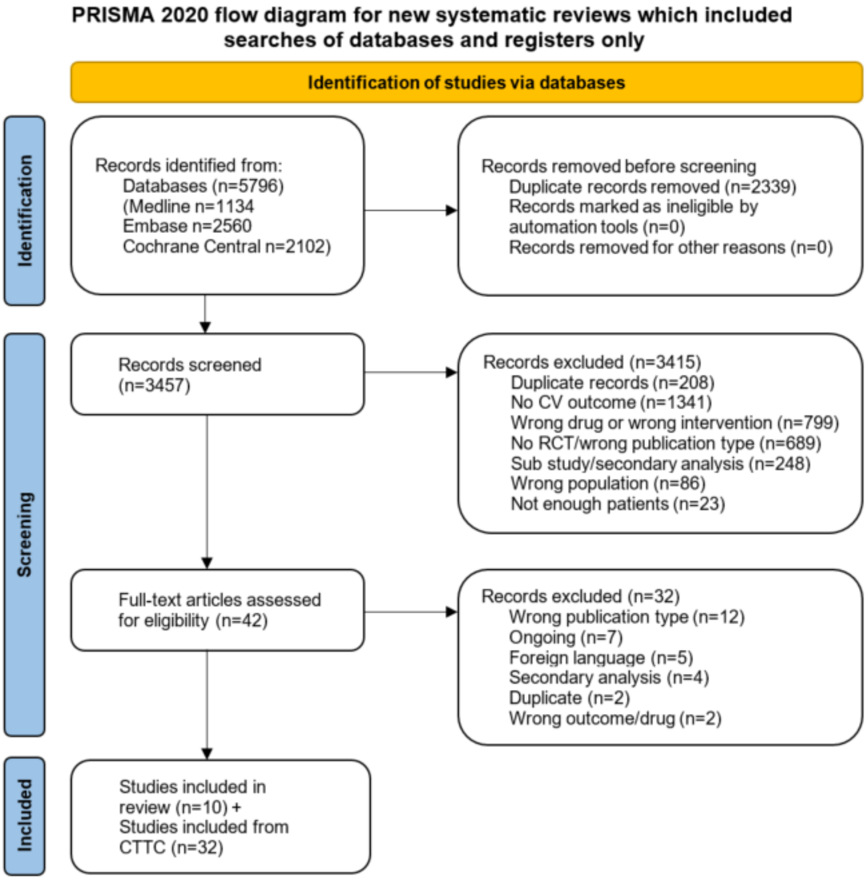

Figure S2: Risk of Bias

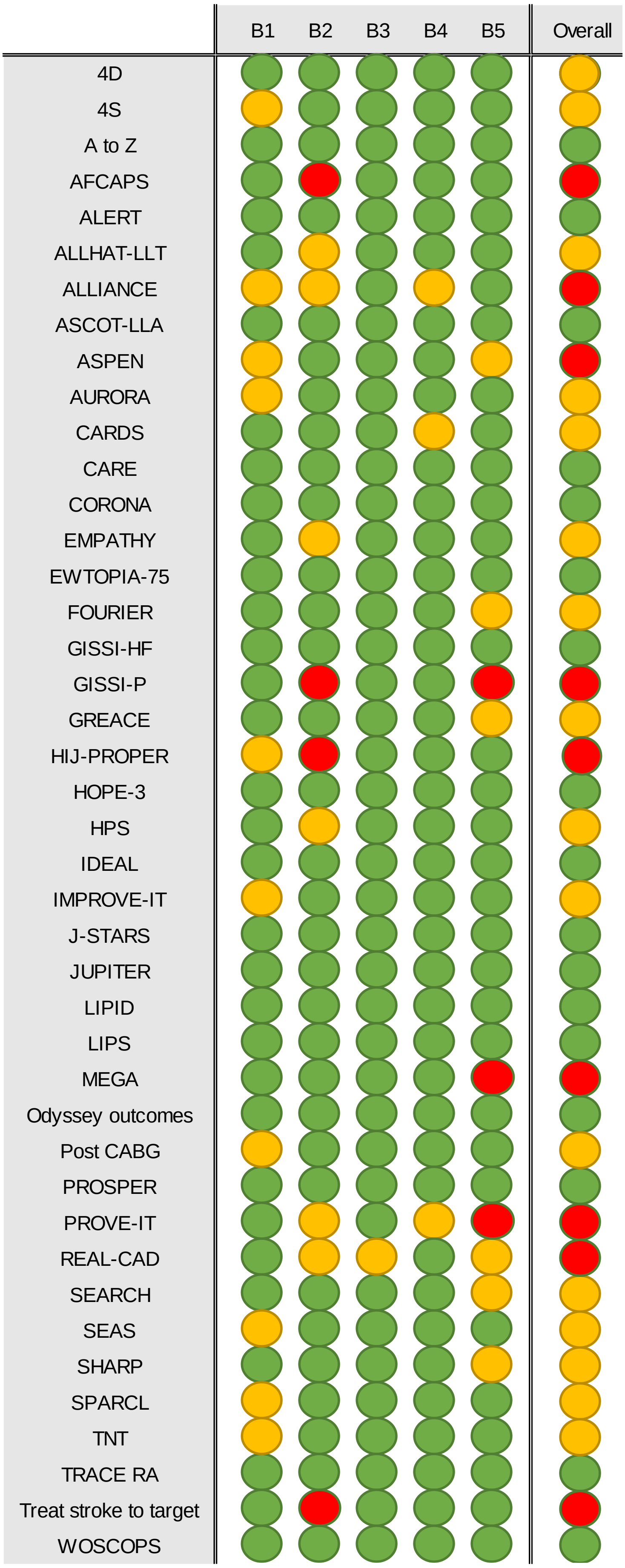

B1: Risk of bias arising from the randomization process

B2: Risk of bias due to deviations from intended interventions

B3: Risk of bias due to missing outcome data

B4: Risk of bias in measurement of the outcome

B5: Risk of bias in selection of the reported result

Figure S3: Percentage of RCTs excluding specific predefined patients' group

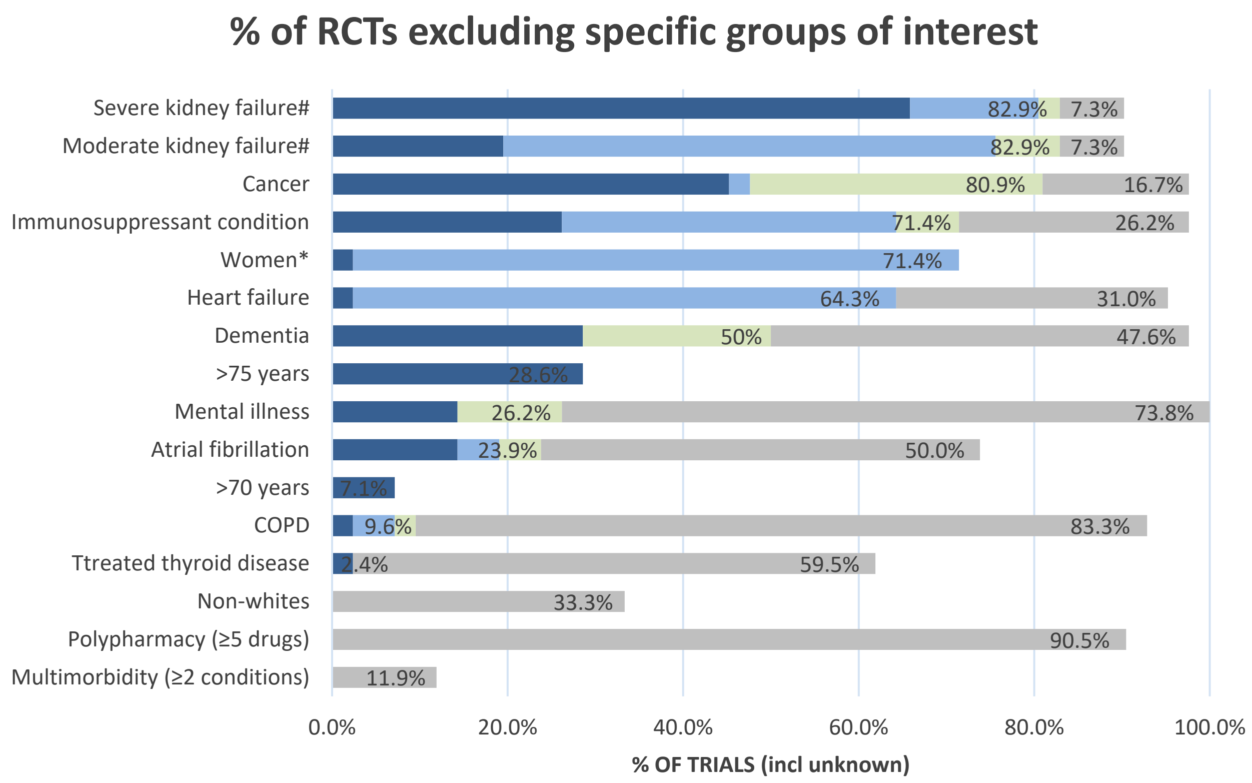

Blue: Patient group as a clearly defined exclusion criterion. % of trials excluding all patients of this exact group. Light blue: Part of patients' group excluded (e.g. only patients with heart failure NYHA III-IV excluded). % of trials excluding a part of this specific patient group. Green: Patients' group not clearly mentioned; medical condition circumscribed. % of trials probably excluding this specific patient group. Grey: Unknown (no information available; mentioned neither as an inclusion nor as an exclusion criterion)

Women*: Group of premenopausal, of childbearing potential, pregnant or lactating women excluded.

Kidney failure#: n=1 trial excluded for analysis (all patients on hemodialysis)

Figure S4: Pooled prevalence of patients above 70 years of age

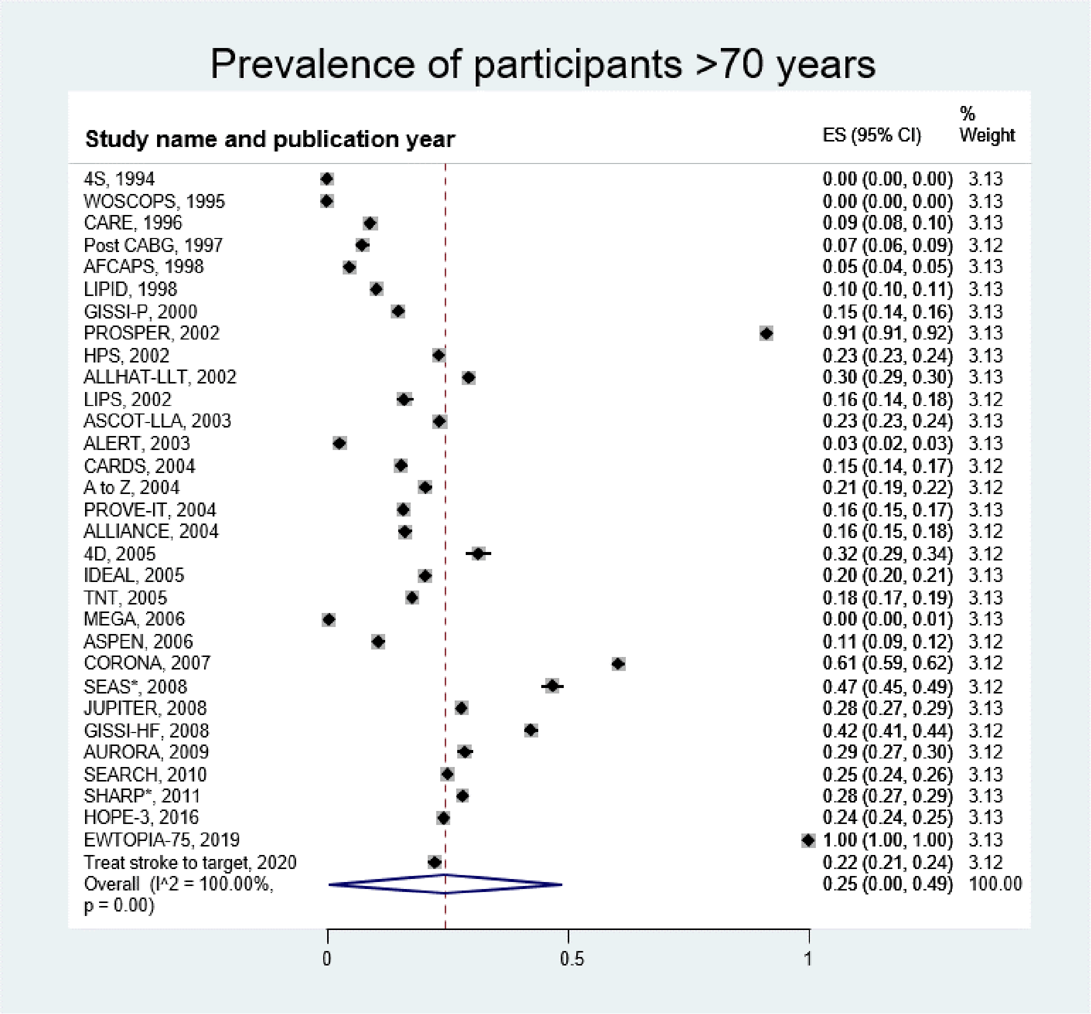

*only data for patients ≥70 years available
